# Crystal structure and Hirshfeld surface analysis of (*Z*)-6-[(2-hy­droxy-5-nitro­anilino)methyl­idene]-4-methyl­cyclo­hexa-2,4-dien-1-one

**DOI:** 10.1107/S205698901900673X

**Published:** 2019-05-17

**Authors:** Sevgi Kansiz, Necmi Dege, Alev Sema Aydin, Erbil Ağar, Igor P. Matushko

**Affiliations:** a Ondokuz Mayıs University, Faculty of Arts and Sciences, Department of Physics, 55139, Kurupelit, Samsun, Turkey; b Ondokuz Mayıs University, Faculty of Arts and Sciences, Department of Physics, 55139, Samsun, Turkey; c Ondokuz Mayıs University, Faculty of Arts and Sciences, Department of Chemistry, 55139, Samsun, Turkey; d Taras Shevchenko National University of Kyiv, Department of Chemistry, 64, Vladimirska Str., Kiev 01601, Ukraine

**Keywords:** crystal structure, Schiff base, hydrogen bonding, Hirshfeld surface analysis

## Abstract

The title compound, C_14_H_12_N_2_O_4_, is a Schiff base that exists in the keto–enamine tautomeric form and adopts a *Z* configuration. The mol­ecule is almost planar, the rings making a dihedral angle of 4.99 (7)°. The mol­ecular structure is stabilized by an intra­molecular N—H⋯O hydrogen bond forming an *S*(6) ring motif. In the crystal, inversion-related mol­ecules are linked by pairs of O—H⋯O hydrogen bonds, forming dimers with an *R* ^2^_2_(18) ring motif. The dimers are linked by pairs of C—H⋯O contacts with an *R* ^2^_2_(10) ring motif, forming ribbons extended along the [20] direction. Hirshfeld surface analysis, two-dimensional fingerprint plots and the mol­ecular electrostatic potential surfaces were used to analyse the inter­molecular inter­actions present in the crystal, indicating that the most important contributions for the crystal packing are from H⋯H (33.9%), O⋯H/H⋯O (29.8%) and C⋯H/H⋯C (17.3%) inter­actions.

## Abstract 2

In the crystal, the mol­ecules are linked by pairs of O—H⋯O hydrogen bonds, forming dimers with an 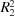
(18) ring motif. The dimers are linked by pairs of C—H⋯O contacts with an 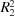
(10) ring motif, forming ribbons extended along the [2
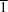
0] direction.

## Chemical context   

Compounds containing the *R*HC=N*R* fragment, obtained by the condensation reaction of primary amines with aldehydes or ketones under proper conditions, are named Schiff bases after Hugo Schiff (Schiff, 1864 ▸). Schiff bases have a wide variety of applications in many areas such as analytical, biological, and inorganic chemistry (Jain *et al.*, 2008 ▸; Lozier *et al.*, 1975 ▸; Calligaris & Randaccio, 1987 ▸). Many Schiff bases are biologically active and some bases show phototochromism which can be used for radiation intensity measurements, display systems or optical devices (Hadjoudis *et al.*, 1987 ▸). In the present study, a new Schiff base, (*Z*)-6-[(2-hy­droxy-5-nitro­anilino)methyl­idene]-4-methyl­cyclo­hexa-2,4-dien-1-one, was obtained in crystalline form from the reaction of 2-amino-4-nitro­phenol with 2-hy­droxy-5-methyl­benzaldehyde. We report here the synthesis and the crystal and mol­ecular structures of the title compound along with the results of a Hirshfeld surface analysis.
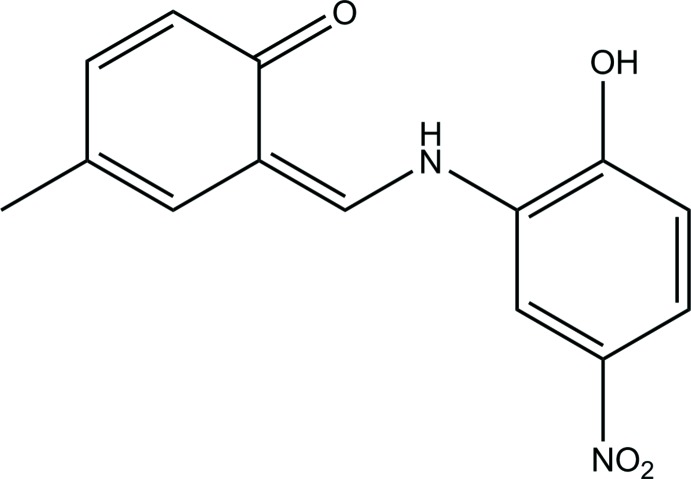


## Structural commentary   

**Figure 1:**
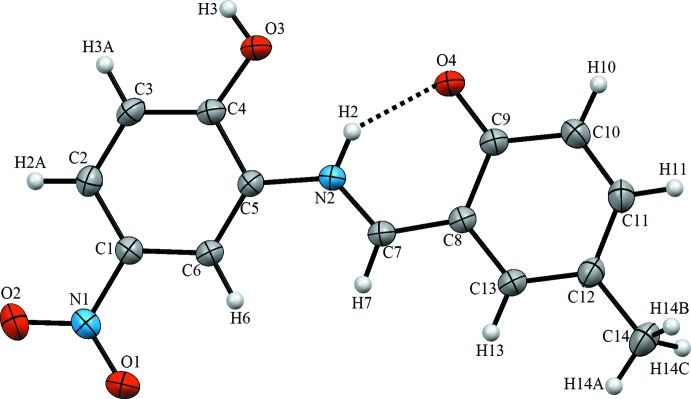
The mol­ecular structure of the title compound, with the atom-labelling scheme. Displacement ellipsoids are drawn at the 40% probability level. Dashed lines denote the intra­molecular N—H⋯O hydrogen bond forming an *S*(6) ring motif.

Fig. 1 ▸ illustrates the mol­ecular structure of the title compound. Its asymmetric unit contains one independent mol­ecule, which adopts the keto–enamine tautomeric form. The mol­ecule is almost planar, the C1–C6 and C8–C13 rings making a dihedral angle of 4.99 (7)°. The O4=C9, C9—C8, C8=C7, C7—N2 and N2—C5 bond lengths are typical of double and single bonds, respectively (Table 1 ▸), thus indicating that the title mol­ecule exists as a keto–enamine tautomer (Kansiz *et al.*, 2018 ▸). The bond lengths at the N1 atom are typical of aromatic nitro groups. The mol­ecular structure is stabilized by the intra­molecular N—H⋯O hydrogen bond involving the keto O4 and amine N2 atoms (Fig. 1 ▸, Table 2 ▸).

## Supra­molecular features   

**Figure 2:**
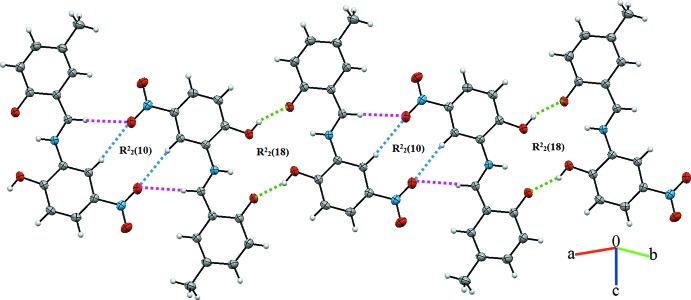
A view of the crystal packing of the title compound. Dashed lines denote the inter­molecular C—H⋯O and O—H⋯O hydrogen bonds forming dimers with 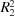
(10) and 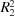
(18) ring motifs (Table 1 ▸).

The most important inter­molecular inter­actions in the title structure are the medium–strong O3—H3⋯O4^i^ hydrogen bonds, which link inversion-related mol­ecules into dimers with an 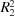
(18) ring motif (Table 2 ▸). These dimers are further connected by pairs of weak C—H⋯O hydrogen bonds with an 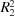
(10) ring motif to form ribbons extended along the [2
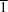
0] direction (Fig. 2 ▸).

## Database survey   

A search of the Cambridge Structural Database (CSD, version 5.40, update November 2018; Groom *et al.*, 2016 ▸) for the 2-[(2-hy­droxy­phenyl­iminio)meth­yl]phenolate fragment revealed 25 hits where this fragment adopts the keto–enamine tautomeric form. The enamine (N2—C7) and keto (C9—O4) bond lengths in the title compound are the same within standard uncertainties as the corresponding bond lengths in the structures of 2-{[(2-hy­droxy­phen­yl)iminio]meth­yl}-4-meth­oxy­phenolate (BALGUR02; Makal *et al.*, 2011 ▸), 4-bromo-2-{[(2-hy­droxy-5-methyl­phen­yl)iminio]meth­yl}phenolate (EYUSIC; Takjoo *et al.*, 2017 ▸), 2-hy­droxy-6-{[(2-hy­droxy­phen­yl)iminio]meth­­yl}phenolate methanol solvate (HEKSIC; Ezeorah *et al.*, 2018 ▸) and 2-{(*E*)-[(5-bromo-2-hy­droxy­phen­yl)methyl­idene]amino}-4-chloro­phenol (SEFKUL; Ebrahimipour *et al.*, 2012 ▸). In the structures of these typical keto–enamine tautomers, the bonds corresponding to C7—C8 in the title structure are distinctly longer, being in the range of 1.416–1.423 Å. As for the C9—O4 bond, its length compares well with 1.286 (2) Å for HEKSIC and 1.291 (2) Å for SEFKUL, but this bond is shorter than 1.298 (2) Å for EYUSIC and 1.310 (2) Å for BALGUR02. It is likely that the inter­molecular O—H⋯O hydrogen bond, where the keto O atom acts as a hydrogen-bond acceptor, is an important prerequisite for the tautomeric shift toward the keto–enamine form. In fact, in all 25 structures of the keto–enamine tautomers, hydrogen bonds of this type are observed.

## Hirshfeld surface analysis   

**Figure 3:**
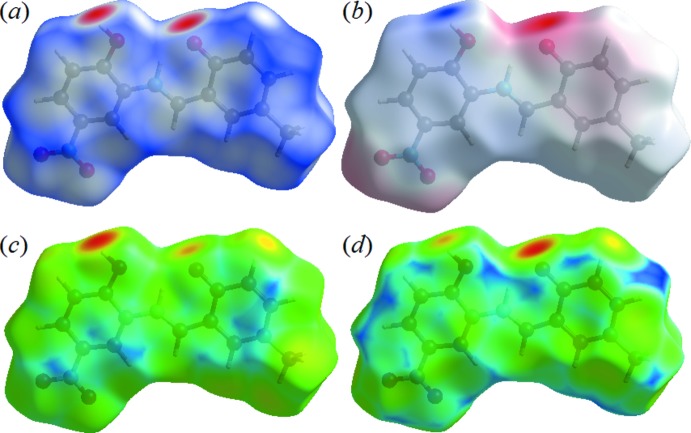
The Hirshfeld surfaces of the title compound mapped over (*a*) *d*_norm_, (*b*) electrostatic potential, (*c*) *d*_i_ and (*d*) *d*_e_.

**Figure 4:**
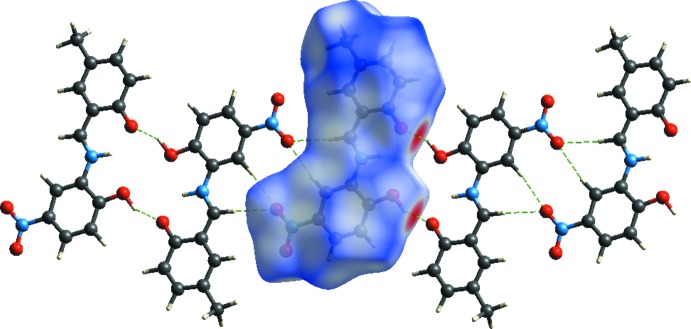
*d*_norm_ mapped on the Hirshfeld surfaces to visualize the inter­molecular inter­actions for the title compound.

The Hirshfeld surface analysis (Spackman & Jayatilaka, 2009 ▸) and the associated two-dimensional fingerprint plots (McKinnon *et al.*, 2007 ▸) were performed with *CrystalExplorer17* (Turner *et al.*, 2017 ▸). The Hirshfeld surfaces were mapped with different properties namely, *d*_norm_, electrostatic potential, *d*_i_ and *d*_e_ (Fig. 3 ▸). The Hirshfeld surfaces mapped over *d_norm_* utilize the function of normalized distances *d*_e_ and *d*_i_, where *d*_e_ and *d*_i_ are the distances from a given point on the surface to the nearest atom outside and inside, respectively. The blue, white and red colour conventions used for the *d*_norm_-mapped Hirshfeld surfaces recognize long inter­molecular contacts, the contacts at the van der Waals separations, and short inter­molecular contacts, respectively. The red region is apparent around the keto oxygen atom (O4) participating in the O—H⋯O and N—H⋯O contacts mentioned above (Fig. 3 ▸, Table 2 ▸). Fig. 4 ▸ illustrates the Hirshfeld surface of the mol­ecule in the crystal, with the evident hydrogen-bonding inter­actions indicated as intense red spots.

**Figure 5:**
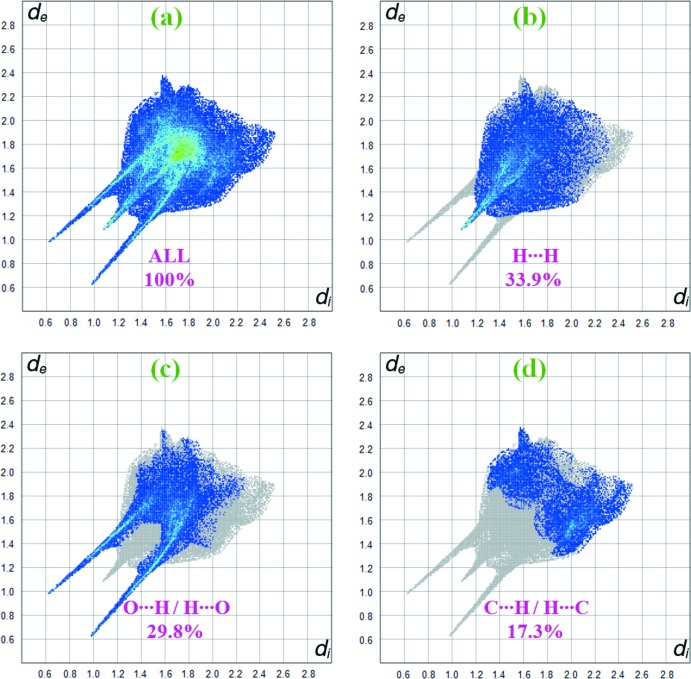
Two-dimensional fingerprint plots for the title compound, with a *d*_norm_ view and the relative contributions of the atom pairs to the Hirshfeld surface.

The two-dimensional fingerprint plot derived from a Hirshfeld surface provides a convenient visual summary of the frequency of each combination of *d*_e_ and *d*_i_ across the surface of a mol­ecule. A fingerprint plot delineated into specific inter­atomic contacts contains information related to specific inter­molecular inter­actions (Tan *et al.*, 2019 ▸). The blue colour refers to the frequency of occurrence of the (*d*_i_, *d*_e_) pairs with the full fingerprint outlined in grey. Fig. 5 ▸ *a* shows the two-dimensional fingerprint of the sum of the contacts contributing to the Hirshfeld surface represented in normal mode. Individual fingerprint plots (Fig. 5 ▸ *b*) reveal that the H⋯H contacts clearly make the most significant contribution to the Hirshfeld surface (33.9%). It is usually the case that the main contribution to the overall surface arises from H⋯H contacts. In addition, O⋯H/H⋯O and C⋯H/H⋯C contacts contribute 29.8% and 17.3%, respectively, to the Hirshfeld surface. In particular, the O⋯H/H⋯O and C⋯H/H⋯C contacts indicate the presence of inter­molecular O—H⋯O and C—H⋯O inter­actions, respectively. Much weaker C⋯O/O⋯C (6.8%) and C⋯C (4.8%) contacts also occur.

**Figure 6:**
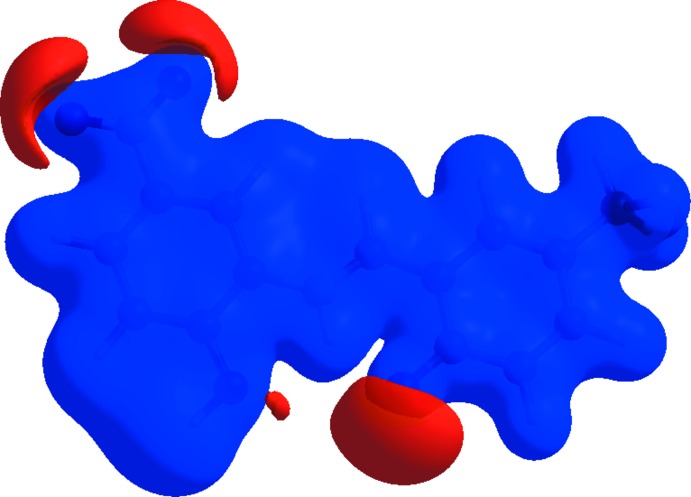
A view of the mol­ecular electrostatic potential of the title compound in the range −0.0500 to 0.0500 a.u. calculated at the HF/STO-3 G level.

The view of the electrostatic potential obtained using *CrystalExplorer17* enables the visualization of the donors and acceptors of inter­molecular inter­actions through blue and red regions around the participating atoms corresponding to positive and negative electrostatic potential, respectively, on the surface. The view of the electrostatic potential in the range −0.0500 to 0.0500 a.u., calculated for the title compound at the HF/STO-3G level, is shown in Fig. 6 ▸. The acceptors for N—H⋯O and O—H⋯O hydrogen bonds are shown as red areas around the O4 atom related with negative electrostatic potentials (Fig. 6 ▸).

## Synthesis and crystallization   

The title compound was prepared by mixing the solutions of 2-hy­droxy-5-methyl­benzaldehyde (34.0 mg, 0.25 mmol) in ethanol (15 ml) and 2-amino-4-nitro­phenol (38.5 mg, 0.25 mmol) in ethanol (15 ml) with subsequent stirring for 5 h under reflux. Single crystals of the title compound suitable for X-ray analysis were obtained by slow evaporation of an ethanol solution (yield 65%, m.p. 523–525 K).

## Refinement   

Crystal data, data collection and structure refinement details are summarized in Table 3 ▸. The hy­droxy H atom was located in a difference-Fourier map, and the OH group was allowed to rotate during the refinement procedure (AFIX 147). The C-bound H atoms were positioned geometrically and refined using a riding model: C—H = 0.93 Å with *U*_iso_(H) = 1.2*U*_eq_(C) for aromatic H atoms and C—H = 0.96 Å with *U*_iso_(H) = 1.5*U*_eq_(C) for methyl H atoms. The amine H atom was also refined using a riding model: N—H = 0.86 Å with *U*_iso_(H) = 1.2*U*_eq_(N).

## Supplementary Material

Crystal structure: contains datablock(s) I. DOI: 10.1107/S205698901900673X/yk2122sup1.cif

Structure factors: contains datablock(s) I. DOI: 10.1107/S205698901900673X/yk2122Isup2.hkl

Supporting information file. DOI: 10.1107/S205698901900673X/yk2122Isup3.cml

CCDC reference: 1915380

Additional supporting information:  crystallographic information; 3D view; checkCIF report

## Figures and Tables

**Table 1:** Selected bond lengths (Å)

O3—C4	1.3329 (14)	O2—N1	1.2213 (15)
O4—C9	1.2887 (15)	N1—C1	1.4530 (15)
N2—C7	1.3070 (15)	C7—C8	1.4054 (16)
N2—C5	1.4035 (15)	C8—C9	1.4340 (15)
O1—N1	1.2202 (15)		

**Table 2:** Hydrogen-bond geometry (Å, °)

*D*—H⋯*A*	*D*—H	H⋯*A*	*D*⋯*A*	*D*—H⋯*A*
N2—H2⋯O4	0.86	1.86	2.5661 (13)	139
O3—H3⋯O4^i^	0.82	1.77	2.5465 (12)	156
C6—H6⋯O1^ii^	0.93	2.51	3.4383 (16)	172
C7—H7⋯O1^ii^	0.93	2.40	3.3182 (14)	172

Symmetry codes: (i) 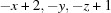; (ii) 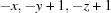.

**Table 3:** Experimental details

Crystal data	
Chemical formula	C_14_H_12_N_2_O_4_
*M*_r_	272.26
Crystal system, space group	Monoclinic, *P*2_1_/*n*
Temperature (K)	296
*a*, *b*, *c* (Å)	6.0052 (4), 7.8206 (5), 26.2985 (19)
β (°)	90.303 (5)
*V* (Å^3^)	1235.07 (14)
*Z*	4
Radiation type	Mo *K*α
μ (mm^−1^)	0.11
Crystal size (mm)	0.57 × 0.43 × 0.19

Data collection	
Diffractometer	Stoe IPDS 2
Absorption correction	Integration (*X-RED32*; Stoe & Cie, 2002 ▸)
*T*_min_, *T*_max_	0.946, 0.981
No. of measured, independent and observed [*I* > 2σ(*I*)] reflections	8536, 3300, 2436
*R*_int_	0.028
(sin θ/λ)_max_ (Å^−1^)	0.686

Refinement	
*R*[*F* ^2^ > 2σ(*F* ^2^)], *wR*(*F* ^2^), *S*	0.041, 0.121, 1.08
No. of reflections	3300
No. of parameters	183
H-atom treatment	H-atom parameters constrained
Δρ_max_, Δρ_min_ (e Å^−3^)	0.15, −0.19

Computer programs: *X-AREA* and *X-RED* (Stoe & Cie, 2002 ▸), *SHELXT2017* (Sheldrick, 2015*a* ▸), *WinGX* (Farrugia, 2012 ▸), *SHELXL2018* (Sheldrick, 2015*b* ▸), *PLATON* (Spek, 2009 ▸) and *publCIF* (Westrip, 2010 ▸).

## supplementary crystallographic information

### Crystal data

**Table:**

C_14_H_12_N_2_O_4_	*F*(000) = 568
*M**_r_* = 272.26	*D*_x_ = 1.464 Mg m^−^^3^
Monoclinic, *P*2_1_/*n*	Mo *K*α radiation, λ = 0.71073 Å
*a* = 6.0052 (4) Å	Cell parameters from 9358 reflections
*b* = 7.8206 (5) Å	θ = 2.6–29.6°
*c* = 26.2985 (19) Å	µ = 0.11 mm^−^^1^
β = 90.303 (5)°	*T* = 296 K
*V* = 1235.07 (14) Å^3^	Plate, orange
*Z* = 4	0.57 × 0.43 × 0.19 mm

### Data collection

**Table:**

STOE IPDS 2 diffractometer	3300 independent reflections
Radiation source: sealed X-ray tube, 12 x 0.4 mm long-fine focus	2436 reflections with *I* > 2σ(*I*)
Detector resolution: 6.67 pixels mm^-1^	*R*_int_ = 0.028
rotation method scans	θ_max_ = 29.2°, θ_min_ = 2.7°
Absorption correction: integration (X-RED32; Stoe & Cie, 2002)	*h* = −7→8
*T*_min_ = 0.946, *T*_max_ = 0.981	*k* = −10→10
8536 measured reflections	*l* = −27→36

### Refinement

**Table:**

Refinement on *F*^2^	0 restraints
Least-squares matrix: full	Hydrogen site location: inferred from neighbouring sites
*R*[*F*^2^ > 2σ(*F*^2^)] = 0.041	H-atom parameters constrained
*wR*(*F*^2^) = 0.121	*w* = 1/[σ^2^(*F*_o_^2^) + (0.0685*P*)^2^ + 0.0139*P*] where *P* = (*F*_o_^2^ + 2*F*_c_^2^)/3
*S* = 1.08	(Δ/σ)_max_ < 0.001
3300 reflections	Δρ_max_ = 0.15 e Å^−^^3^
183 parameters	Δρ_min_ = −0.19 e Å^−^^3^

### Special details

**Table:**

Geometry. All esds (except the esd in the dihedral angle between two l.s. planes) are estimated using the full covariance matrix. The cell esds are taken into account individually in the estimation of esds in distances, angles and torsion angles; correlations between esds in cell parameters are only used when they are defined by crystal symmetry. An approximate (isotropic) treatment of cell esds is used for estimating esds involving l.s. planes.

### Fractional atomic coordinates and isotropic or equivalent isotropic displacement parameters (Å^2^)

**Table:**

	*x*	*y*	*z*	*U*_iso_*/*U*_eq_	
O3	0.85290 (16)	0.10060 (14)	0.53689 (4)	0.0593 (3)	
H3	0.926262	0.063519	0.560850	0.089*	
O4	0.85793 (15)	0.05497 (13)	0.40914 (4)	0.0573 (3)	
N2	0.57371 (16)	0.20069 (12)	0.46738 (4)	0.0413 (2)	
H2	0.697995	0.148587	0.462636	0.050*	
O1	0.00525 (18)	0.53706 (15)	0.56524 (4)	0.0687 (3)	
O2	0.0910 (2)	0.50110 (16)	0.64372 (4)	0.0716 (3)	
N1	0.12631 (18)	0.47944 (14)	0.59848 (4)	0.0482 (3)	
C5	0.52750 (19)	0.24767 (14)	0.51773 (4)	0.0377 (2)	
C1	0.32008 (19)	0.38097 (14)	0.58331 (5)	0.0400 (3)	
C6	0.34505 (19)	0.34263 (14)	0.53221 (4)	0.0394 (2)	
H6	0.241371	0.379997	0.508283	0.047*	
C7	0.45170 (19)	0.22613 (14)	0.42678 (4)	0.0405 (3)	
H7	0.315808	0.281942	0.430109	0.049*	
C4	0.68303 (19)	0.19257 (15)	0.55447 (5)	0.0423 (3)	
C8	0.51880 (18)	0.17203 (15)	0.37826 (4)	0.0397 (2)	
C13	0.3761 (2)	0.20126 (16)	0.33610 (5)	0.0440 (3)	
H13	0.242313	0.258453	0.341190	0.053*	
C9	0.72607 (19)	0.08425 (16)	0.37131 (5)	0.0432 (3)	
C12	0.4290 (2)	0.14798 (17)	0.28810 (5)	0.0476 (3)	
C2	0.4709 (2)	0.32843 (16)	0.61998 (5)	0.0457 (3)	
H2A	0.450177	0.356630	0.653984	0.055*	
C3	0.6520 (2)	0.23381 (16)	0.60524 (5)	0.0480 (3)	
H3A	0.754588	0.197052	0.629487	0.058*	
C11	0.6332 (2)	0.06220 (19)	0.28190 (5)	0.0541 (3)	
H11	0.672078	0.024806	0.249571	0.065*	
C10	0.7763 (2)	0.03164 (19)	0.32125 (5)	0.0540 (3)	
H10	0.909625	−0.024955	0.315049	0.065*	
C14	0.2767 (3)	0.1771 (2)	0.24318 (5)	0.0638 (4)	
H14A	0.141128	0.229334	0.254508	0.096*	
H14B	0.243485	0.069530	0.227267	0.096*	
H14C	0.348636	0.250904	0.219161	0.096*	

### Atomic displacement parameters (Å^2^)

**Table:**

	*U*^11^	*U*^22^	*U*^33^	*U*^12^	*U*^13^	*U*^23^
O3	0.0503 (5)	0.0818 (6)	0.0458 (5)	0.0315 (5)	−0.0095 (4)	−0.0046 (5)
O4	0.0477 (5)	0.0811 (6)	0.0430 (5)	0.0271 (5)	−0.0075 (4)	−0.0024 (4)
N2	0.0384 (5)	0.0503 (5)	0.0352 (5)	0.0132 (4)	−0.0015 (4)	−0.0015 (4)
O1	0.0591 (6)	0.0891 (7)	0.0578 (6)	0.0363 (5)	−0.0010 (5)	−0.0009 (5)
O2	0.0699 (7)	0.0984 (8)	0.0467 (6)	0.0202 (6)	0.0138 (5)	−0.0104 (5)
N1	0.0454 (6)	0.0538 (6)	0.0455 (6)	0.0084 (5)	0.0058 (5)	−0.0033 (5)
C5	0.0383 (5)	0.0422 (5)	0.0327 (5)	0.0055 (4)	−0.0029 (4)	−0.0001 (4)
C1	0.0389 (6)	0.0429 (6)	0.0382 (6)	0.0042 (4)	0.0009 (4)	−0.0006 (4)
C6	0.0375 (5)	0.0456 (6)	0.0351 (6)	0.0068 (4)	−0.0033 (4)	0.0022 (4)
C7	0.0386 (5)	0.0459 (6)	0.0369 (6)	0.0103 (4)	−0.0031 (4)	−0.0002 (4)
C4	0.0379 (5)	0.0479 (6)	0.0409 (6)	0.0088 (5)	−0.0056 (5)	−0.0004 (5)
C8	0.0392 (6)	0.0443 (6)	0.0356 (5)	0.0068 (4)	−0.0014 (4)	0.0011 (4)
C13	0.0416 (6)	0.0527 (6)	0.0378 (6)	0.0091 (5)	−0.0042 (5)	0.0025 (5)
C9	0.0390 (6)	0.0521 (6)	0.0384 (6)	0.0080 (5)	−0.0023 (5)	0.0011 (5)
C12	0.0492 (7)	0.0590 (7)	0.0344 (6)	0.0014 (5)	−0.0037 (5)	0.0033 (5)
C2	0.0513 (7)	0.0517 (6)	0.0339 (6)	0.0043 (5)	−0.0029 (5)	−0.0021 (5)
C3	0.0477 (6)	0.0579 (7)	0.0382 (6)	0.0091 (5)	−0.0125 (5)	0.0007 (5)
C11	0.0537 (7)	0.0733 (9)	0.0354 (6)	0.0060 (6)	0.0043 (5)	−0.0040 (6)
C10	0.0453 (7)	0.0722 (8)	0.0445 (7)	0.0148 (6)	0.0044 (5)	−0.0040 (6)
C14	0.0669 (9)	0.0863 (10)	0.0381 (7)	0.0072 (8)	−0.0102 (6)	0.0039 (7)

### Geometric parameters (Å, º)

**Table:**

O3—C4	1.3329 (14)	C8—C13	1.4162 (16)
O3—H3	0.8200	C8—C9	1.4340 (15)
O4—C9	1.2887 (15)	C13—C12	1.3683 (18)
N2—C7	1.3070 (15)	C13—H13	0.9300
N2—C5	1.4035 (15)	C9—C10	1.4134 (18)
N2—H2	0.8600	C12—C11	1.4077 (19)
O1—N1	1.2202 (15)	C12—C14	1.5074 (19)
O2—N1	1.2213 (15)	C2—C3	1.3726 (17)
N1—C1	1.4530 (15)	C2—H2A	0.9300
C5—C6	1.3789 (15)	C3—H3A	0.9300
C5—C4	1.4077 (15)	C11—C10	1.3629 (19)
C1—C2	1.3823 (17)	C11—H11	0.9300
C1—C6	1.3857 (16)	C10—H10	0.9300
C6—H6	0.9300	C14—H14A	0.9600
C7—C8	1.4054 (16)	C14—H14B	0.9600
C7—H7	0.9300	C14—H14C	0.9600
C4—C3	1.3873 (18)		
			
C4—O3—H3	109.5	C12—C13—H13	119.0
C7—N2—C5	128.19 (10)	C8—C13—H13	119.0
C7—N2—H2	115.9	O4—C9—C10	122.28 (11)
C5—N2—H2	115.9	O4—C9—C8	121.13 (11)
O1—N1—O2	122.72 (11)	C10—C9—C8	116.59 (11)
O1—N1—C1	118.29 (10)	C13—C12—C11	117.30 (12)
O2—N1—C1	118.99 (11)	C13—C12—C14	122.28 (12)
C6—C5—N2	124.25 (10)	C11—C12—C14	120.42 (12)
C6—C5—C4	120.05 (10)	C3—C2—C1	118.70 (11)
N2—C5—C4	115.70 (10)	C3—C2—H2A	120.6
C2—C1—C6	122.58 (11)	C1—C2—H2A	120.6
C2—C1—N1	119.25 (11)	C2—C3—C4	120.53 (11)
C6—C1—N1	118.17 (10)	C2—C3—H3A	119.7
C5—C6—C1	118.33 (10)	C4—C3—H3A	119.7
C5—C6—H6	120.8	C10—C11—C12	122.80 (12)
C1—C6—H6	120.8	C10—C11—H11	118.6
N2—C7—C8	122.26 (10)	C12—C11—H11	118.6
N2—C7—H7	118.9	C11—C10—C9	121.30 (12)
C8—C7—H7	118.9	C11—C10—H10	119.4
O3—C4—C3	124.50 (10)	C9—C10—H10	119.4
O3—C4—C5	115.68 (10)	C12—C14—H14A	109.5
C3—C4—C5	119.81 (10)	C12—C14—H14B	109.5
C7—C8—C13	119.15 (10)	H14A—C14—H14B	109.5
C7—C8—C9	120.86 (10)	C12—C14—H14C	109.5
C13—C8—C9	119.97 (10)	H14A—C14—H14C	109.5
C12—C13—C8	122.04 (11)	H14B—C14—H14C	109.5
			
C7—N2—C5—C6	−4.9 (2)	C9—C8—C13—C12	0.05 (19)
C7—N2—C5—C4	175.61 (12)	C7—C8—C9—O4	−1.11 (19)
O1—N1—C1—C2	173.86 (12)	C13—C8—C9—O4	−179.47 (12)
O2—N1—C1—C2	−6.20 (19)	C7—C8—C9—C10	178.60 (12)
O1—N1—C1—C6	−6.51 (18)	C13—C8—C9—C10	0.25 (18)
O2—N1—C1—C6	173.42 (12)	C8—C13—C12—C11	−0.1 (2)
N2—C5—C6—C1	−179.62 (11)	C8—C13—C12—C14	179.34 (13)
C4—C5—C6—C1	−0.16 (17)	C6—C1—C2—C3	−0.3 (2)
C2—C1—C6—C5	0.28 (18)	N1—C1—C2—C3	179.26 (11)
N1—C1—C6—C5	−179.33 (10)	C1—C2—C3—C4	0.3 (2)
C5—N2—C7—C8	179.95 (11)	O3—C4—C3—C2	−179.58 (13)
C6—C5—C4—O3	179.56 (11)	C5—C4—C3—C2	−0.2 (2)
N2—C5—C4—O3	−0.93 (17)	C13—C12—C11—C10	−0.1 (2)
C6—C5—C4—C3	0.12 (18)	C14—C12—C11—C10	−179.58 (15)
N2—C5—C4—C3	179.62 (11)	C12—C11—C10—C9	0.4 (2)
N2—C7—C8—C13	178.88 (11)	O4—C9—C10—C11	179.23 (13)
N2—C7—C8—C9	0.51 (19)	C8—C9—C10—C11	−0.5 (2)
C7—C8—C13—C12	−178.33 (12)		

### Hydrogen-bond geometry (Å, º)

**Table:**

*D*—H···*A*	*D*—H	H···*A*	*D*···*A*	*D*—H···*A*
N2—H2···O4	0.86	1.86	2.5661 (13)	139
O3—H3···O4^i^	0.82	1.77	2.5465 (12)	156
C6—H6···O1^ii^	0.93	2.51	3.4383 (16)	172
C7—H7···O1^ii^	0.93	2.40	3.3182 (14)	172

Symmetry codes: (i) −*x*+2, −*y*, −*z*+1; (ii) −*x*, −*y*+1, −*z*+1.
